# Generative Artificial Intelligence Enhancements for Reducing Image-based Training Data Requirements

**DOI:** 10.1016/j.xops.2024.100531

**Published:** 2024-04-14

**Authors:** Dake Chen, Ying Han, Jacque Duncan, Lin Jia, Jing Shan

**Affiliations:** 1 Department of Ophthalmology, University of California, San Francisco, San Francisco, California; 2 Digillect LLC, San Francisco, California

**Keywords:** Glaucoma, Generative AI, Data scarcity

## Abstract

**Objective:**

Training data fuel and shape the development of artificial intelligence (AI) models. Intensive data requirements are a major bottleneck limiting the success of AI tools in sectors with inherently scarce data. In health care, training data are difficult to curate, triggering growing concerns that the current lack of access to health care by under-privileged social groups will translate into future bias in health care AIs. In this report, we developed an autoencoder to grow and enhance inherently scarce datasets to alleviate our dependence on big data.

**Design:**

Computational study with open-source data.

**Subjects:**

The data were obtained from 6 open-source datasets comprising patients aged 40–80 years in Singapore, China, India, and Spain.

**Methods:**

The reported framework generates synthetic images based on real-world patient imaging data. As a test case, we used autoencoder to expand publicly available training sets of optic disc photos, and evaluated the ability of the resultant datasets to train AI models in the detection of glaucomatous optic neuropathy.

**Main Outcome Measures:**

Area under the receiver operating characteristic curve (AUC) were used to evaluate the performance of the glaucoma detector. A higher AUC indicates better detection performance.

**Results:**

Results show that enhancing datasets with synthetic images generated by autoencoder led to superior training sets that improved the performance of AI models.

**Conclusions:**

Our findings here help address the increasingly untenable data volume and quality requirements for AI model development and have implications beyond health care, toward empowering AI adoption for all similarly data-challenged fields.

**Financial Disclosure(s):**

The authors have no proprietary or commercial interest in any materials discussed in this article.

Glaucoma is a leading cause of irreversible blindness, affecting >75 million people worldwide in 2020 and is projected to increase to >111 million by 2040.^1^ Early detection and treatment are key to preserving vision^2^; however, this is challenging because of the often asymptomatic nature of early glaucoma, as much as 50% of glaucoma cases in developed countries remain undetected, whereas, in resource-poor, underdeveloped countries, up to 90% of individuals with glaucoma are undiagnosed.^3^ Furthermore, the increasing prevalence of glaucoma from our aging populations is leading to a growing discrepancy between the supply and demand of glaucoma care; and, as glaucoma progresses, the costs of care increases, further hindering the treatment of this blinding condition. Therefore, there is a strong need for more accessible, objective, and high-throughput detection methods for glaucoma.

Deep learning, a branch of artificial intelligence (AI), has demonstrated effectiveness in detecting glaucomatous changes on clinical testing modalities such as optic disc photos, optical coherence tomography, and Humphrey visual fields.^4^, ^5^, ^6^, ^7^, ^8^ Computer vision models such as convolutional neural networks (CNNs), in particular, have the potential to offer objective, quantitative, and high-throughput glaucoma detection capabilities needed for population-based screening.^9^, ^10^, ^11^, ^12^ Recently, a novel deep learning architecture, the vision transformer (ViT), has demonstrated superior performance over CNNs in the detection of glaucoma.^13^ The vision transformer uses the attention mechanism to capture feature dependencies and relationships within an image and has achieved state-of-the-art performance in various computer vision tasks, such as image classification,^14^ object detection,^15^ and semantic segmentation,^16^ all of which are highly relevant to glaucoma detection.

The development and performance of these deep learning models rely heavily on large-scale datasets, with increasingly untenable volume and labeling requirements. Image classification tasks, for instance, require training CNNs or ViTs with >10 million images.^13^^,^^14^ Similarly, achieving state-of-the-art performance in other computer vision tasks like object detection and semantic segmentation need training backbone models with millions of images.^15^, ^16^, ^17^ This poses significant challenges for data-limited fields such as medicine, where data acquisition and labeling are lengthy and resource-intensive and require specialized expertise. As a result, public glaucoma datasets typically consist of only a few hundred to several thousand images.^9^^,^^18^, ^19^, ^20^, ^21^, ^22^ In addition to limiting model performance, this also raises concerns regarding the fairness and equity of AI models in this domain. In the field of deep learning, it is widely accepted that an increase in the amount of data generally results in improved model performance. Underrepresented social groups contribute significantly less health care data,^23^ thus potentially propagating their current lack of access to health care into future bias in health care AIs.

Recent emergence of generative AI technologies has introduced the concept of synthetically enhanced datasets^24^^,^^25^ as a means to increase model performance while decreasing training data requirements. Although exciting, generative AIs suffer from the problem of hallucination,^26^ a phenomenon in which the model outputs are so divorced from reality that the results are nonsensical. Hallucination is believed to arise from a number of factors, including insufficient or biased training data, the very issues we are looking to address. Therefore, to circumvent this active problem, we report instead an autoencoder to produce high-fidelity synthetic images that can be used to improve health care AI model performance and alleviate our debilitating dependence on big data.

## Methods

### Datasets

Original patient fundus photos were sourced from 6 public datasets comprising retinal fundus images for glaucoma detection.^39^, ^40^, ^41^ Each dataset was independently explored and divided into training and validation sets. Because of the relatively small sizes of the datasets, a separate testing set was not allocated. ACRIMA^9^ consists of 705 retinal fundus images, captured from dilated eyes, centered on the optic disc, and selected based on high image quality. Two experienced glaucoma experts annotated the images. Drishti-GS1^19^ was specifically designed for automated glaucoma assessment and comprises 101 fundus images. The patient population for this database spanned 40–80 years of age with roughly equal numbers of men and women. All included images were annotated by glaucoma experts. ORIGA^42^ contains 650 retinal fundus images collected and annotated by the Singapore Eye Research Institute during a 3-year period from 2004 to 2007, involving 149 adult patients, sampled from a larger cross-sectional epidemiological study for visual impairment risk factors among Singapore Malay adults from 40 to 80 years of age. RIM-ONE^21^ comprises 485 retinal fundus images collected from 3 hospitals in different regions of Spanish. Five glaucoma experts from these hospitals provided annotations. The subjects included randomly selected glaucomatous patients and volunteers with healthy eyes. Sjchoi86-HRF^22^ contains fundus images from 4 ophthalmic conditions: normal, cataract, glaucoma, and retinal disease. Only the normal and glaucoma data are used in this study and together a total of 401 fundus images. REFUGE2^18^ provides 800 color fundus photos, captured from both the right and left eyes and annotated by experienced ophthalmologists. All images in this database met the required quality standards.

### Data Preparation

**Figure 1:**
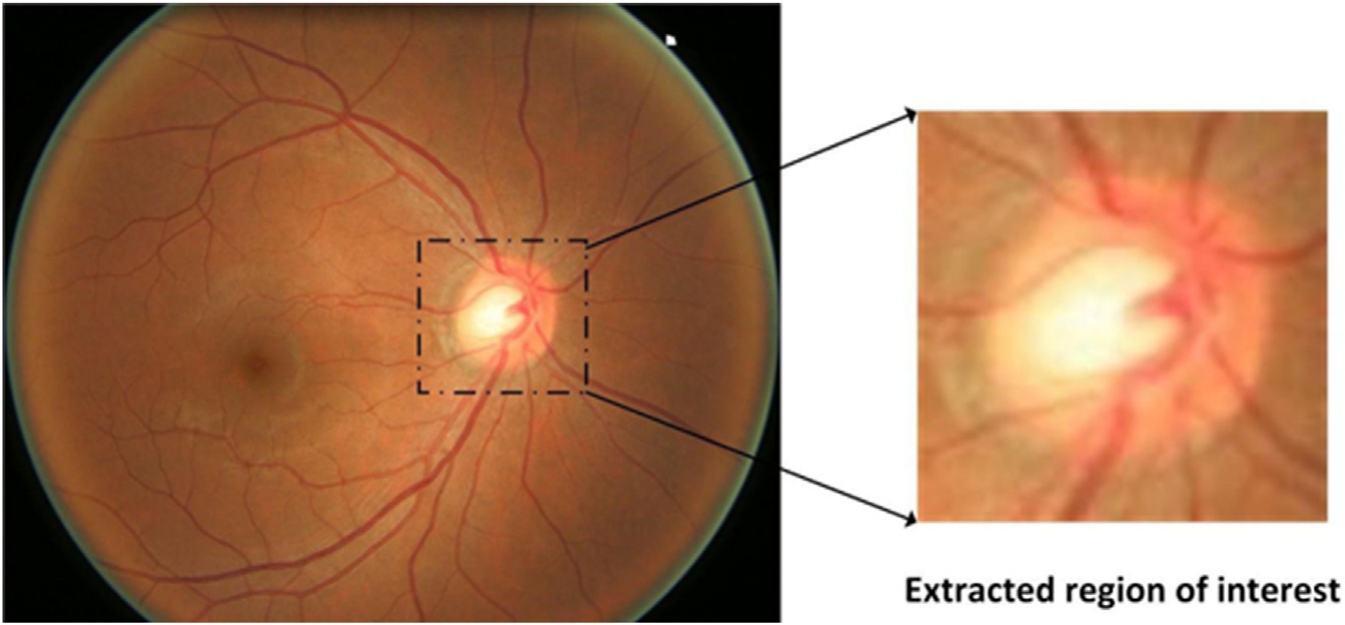
Extraction of region of interest.

To minimize the presence of redundant information, which may negatively impact deep learning model performance, we preprocessed all images to extract the region around the optic nerve head from each fundus image as shown in Figure 1. This was achieved using deeplabv3plus,^43^ a semantic segmentation model. Once the region of interest was extracted, we automatically cropped a square area centered around the disc. These extracted regions of interest were then utilized to train ViTs. By focusing on these specific areas, we aimed to improve the model's ability to identify glaucoma-related features and enhance the accuracy of the automated detection system.

### Autoencoder

**Figure 2:**
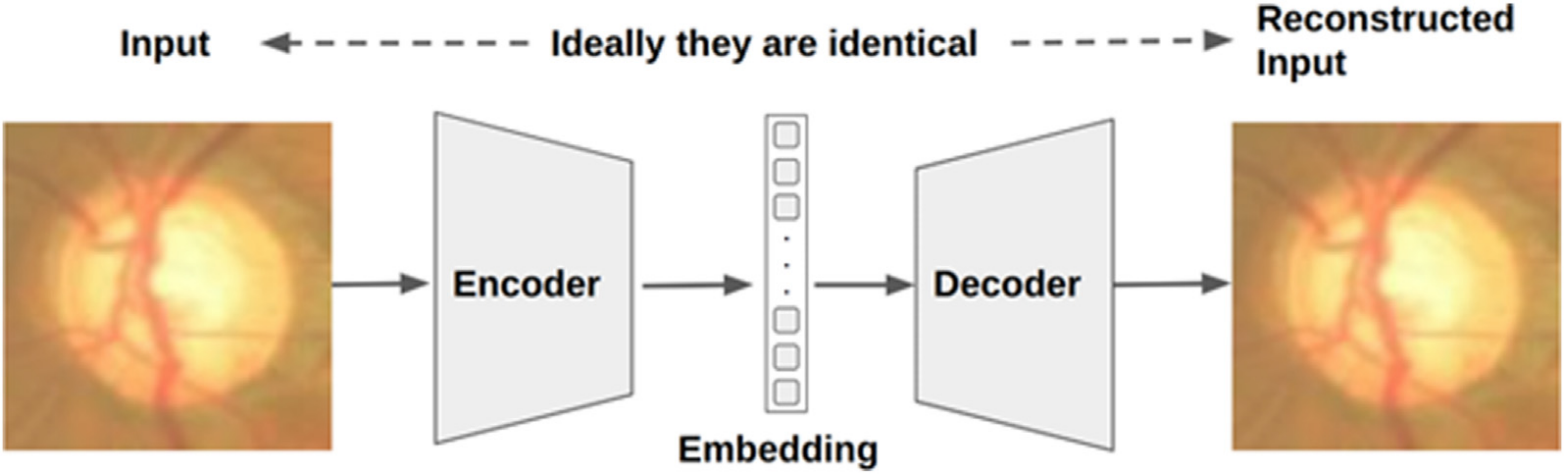
Illustration of the autoencoder architecture.

The autoencoder^28^ is a powerful generative model that has the ability to learn the underlying distribution of data and generate new data samples based on that distribution. As shown in Figure 2, it consists of 2 components: an encoder and a decoder, both of which are neural networks or variants thereof depending on the specific application.

**Figure 3:**
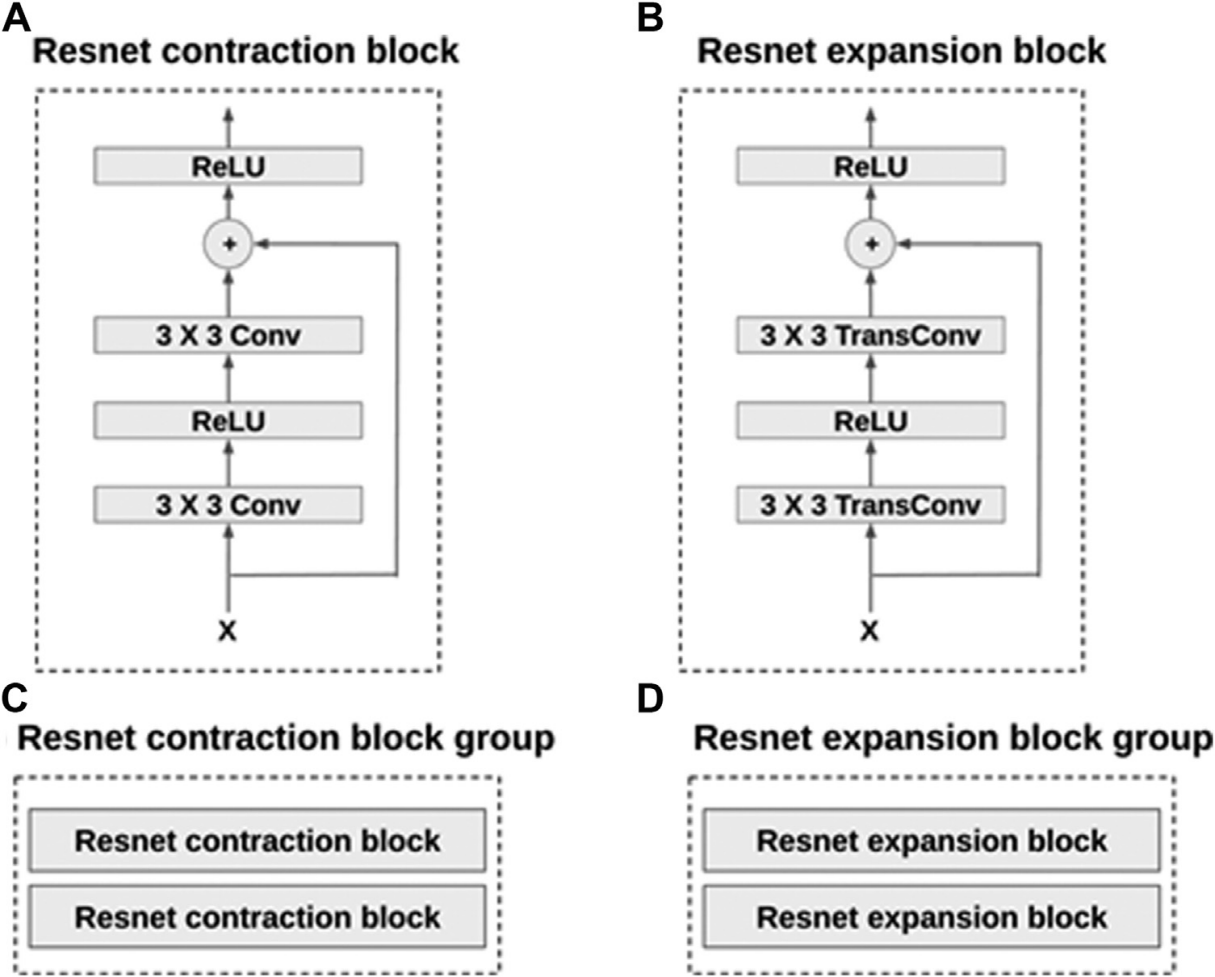
Illustration of the autoencoder building blocks. **A** is the neural net structure of resnet contraction block, and **B** is the structure of resnet expansion block. **C, D** are resnet block groups and each of them consists of 2 blocks.

Our encoder and decoder are designed hierarchically, drawing significant inspiration from ResNet architecture.^29^ They are composed of fundamental building blocks known as ResNet blocks. The encoder utilizes ResNet contraction blocks, whereas the decoder employs ResNet expansion blocks. Figure 3A, B display the neural network architectures of these blocks. These blocks are organized into 2 groups: the ResNet contraction group and the ResNet expansion group, each comprising 2 blocks, as depicted in Figure 3C, D.

**Figure 4:**
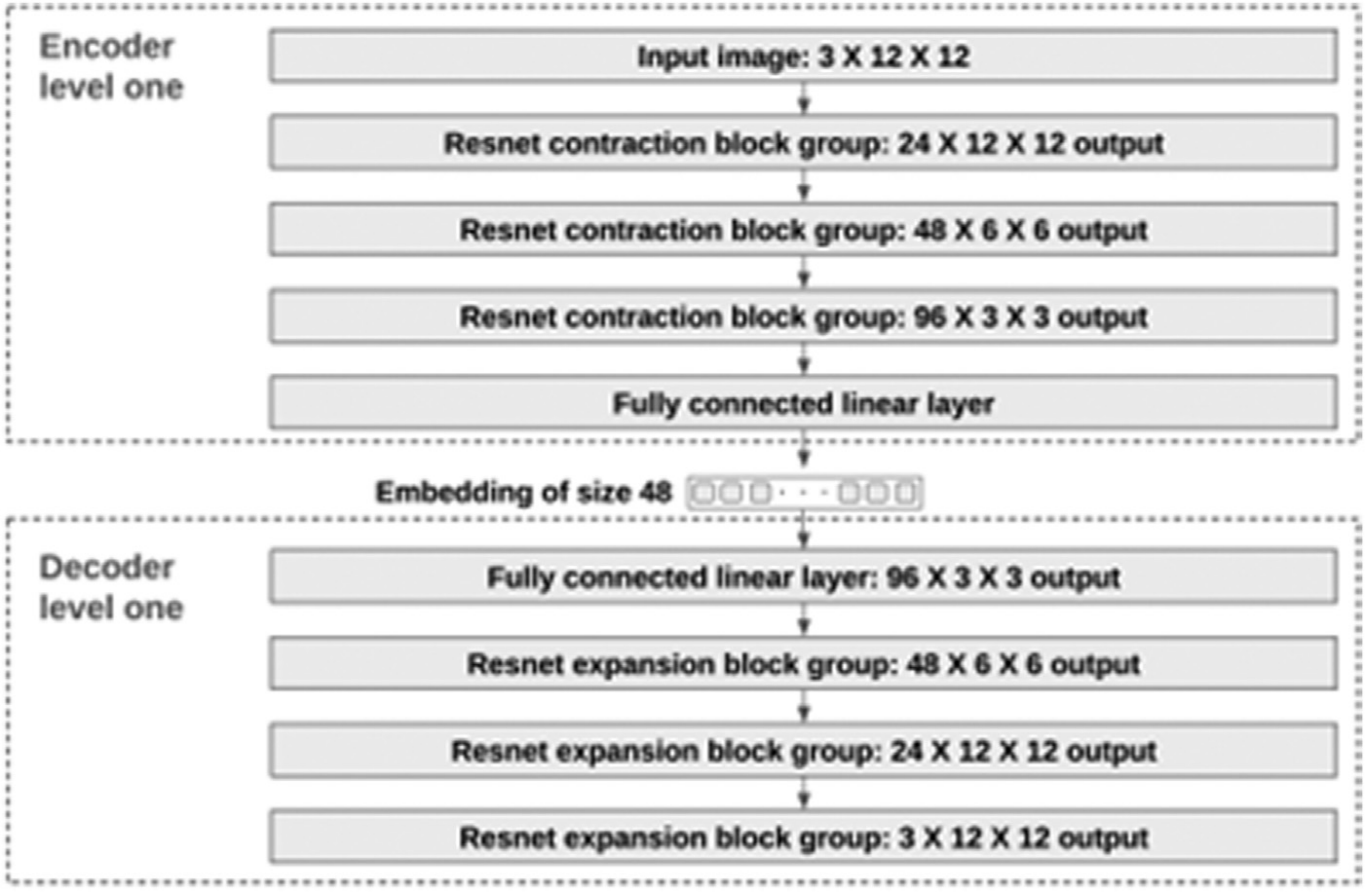
The neural net structure of the level one encoder and decoder.

**Figure 5:**
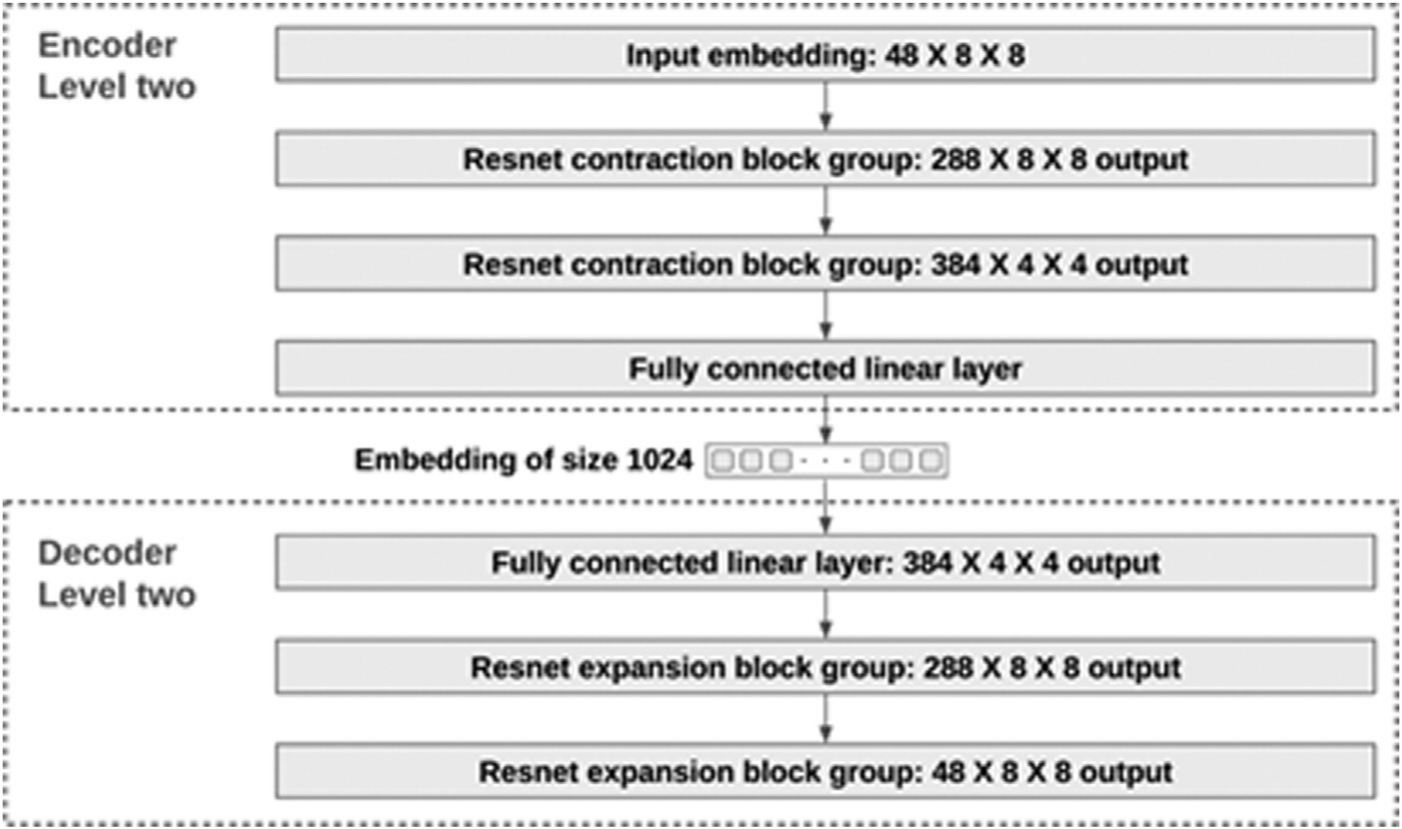
The neural net structure of the level 2 encoder and decoder.

Our autoencoder features a dual-level structure. The Level 1 encoder and decoder are capable of processing images of size 12 × 12, producing an embedding of size 48. Figure 4 shows the neural network architecture of these components. Because most images exceed a 12 × 12 resolution, we segment them into 12 × 12 patches. Each patch is then encoded into a separate embedding for further processing. For instance, a 96 × 96 image can be encoded by the Level 1 encoder into a latent space of dimensions 48 × 8 × 8, where 48 represents the channel size (embedding size). Additionally, we have developed a Level 2 encoder and decoder for processing the latent space, with their architecture detailed in Figure 5.

**Figure 6:**
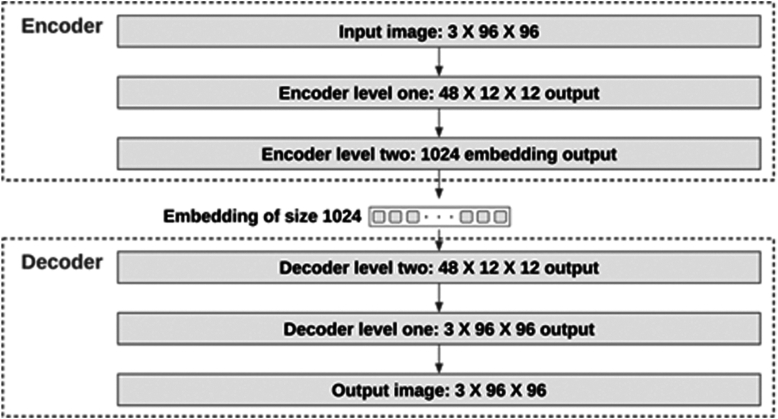
Level 1 and level 2 encoders/decoders can be joined together to encode and decode large image.

The combined functionality of the Level 1 and Level 2 encoders and decoders enables converting a 96 × 96 image into an embedding of size 1024. The entire process is illustrated in Figure 6. This dual-level structure offers several advantages over a single-level system:

a) Training efficiency: by segmenting the encoder and decoder into 2 levels, each can be trained independently, reducing the required GPU memory.
b) Dynamic composition ability: a specific Level 1 encoder can be paired with various Level 2 encoders, allowing for the encoding of images of different sizes into latent of varying dimensions.

In the training of the autoencoder, the employed loss function was defined based on the discrepancy in pixel values between the input image fed into the encoder and the resultant image output from the decoder. The autoencoder framework developed in this study is a general-purpose image autoencoder, capable of encoding and decoding images across a wide spectrum of types, rather than being restricted to images belonging to a specific category. Consequently, the training dataset was selected to encompass as broad a range of images as feasible. We find ImageNet^37^ is highly suitable because of its extensive and diverse collection of images. Given the vast quantity of available training data in ImageNet, the conventional approach of segregating data into training and validation subsets was not employed. Instead, uninterrupted training was conducted. After approximately 200 000 training iterations, the autoencoder demonstrated proficiency in encoding and decoding images beyond those contained within the ImageNet dataset, achieving minimal loss. The specific training hyperparameters are in Table 1.

**Table 1:** Hyperparameters Used for the Training Process of Encoder/Decoder

Hyperparameter	Value
Learning rate	3e-4
Optimizer	AdamW
Weight decay	5e-7
Loss function type	MAE
Activation type	Leaky ReLU
Training batch size	400
Weight precision	fp16

The encoder is trained to map input data to a lower-dimensional latent representation, which is a stochastic compressed representation of the data. The decoder, on the other hand, generates output images from the latent representation using transpose convolutional neural net. The autoencoder deployed for this report was trained with imageNet, a general image data bank, without any additional medical images. Using the trained autoencoder, we generated 3 times the number of training images available in each database and randomly selected one-third of those synthetic images to achieve 100% expansion of the training subset for each database.

### ViT Training and Evaluation

We chose the vision transformer architecture for its outstanding performance in glaucoma detection.^44^ Each of the 6 public databases was split into a training set (80%) and a validation set (20%). The training set alone was used to generate synthetic images using the autoencoder. The vision transformer performance was evaluated using both the original datasets and expanded datasets containing synthetic images. By comparing the performance of the ViTs trained with and without synthetic data, we quantified the specific contribution of the synthetic data in enhancing the model's performance.

To ensure consistency, we standardized the images by resizing them to a uniform size of 224 × 224 pixels. Additionally, we normalized the pixel values to a range between 0 and 1. During training, we used a batch size of 16 and employed the AdamW optimizer with a learning rate of 6e-4 and regularization of 6e-2. These hyperparameters were chosen to optimize the model's convergence and performance. To compute the loss during training, we employed crossentropy loss with 0.1 label smoothing.

The performance of the ViT models was evaluated using the AUC with 95% confidence intervals. The sensitivity and specificity of the system were also reported.

## Results

### Synthetic Image Production

**Figure 7:**
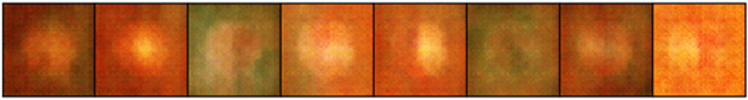
A retinal photograph produced by a Generative Adversarial Network. Ophthalmic evaluation has determined that these images are unsuitable for use in training deep learning models. This finding is in contrast to the effectiveness of the autoencoder approach proposed in this paper, which has been demonstrated to generate synthetic training data of sufficient quality for such applications.

**Figure 8:**
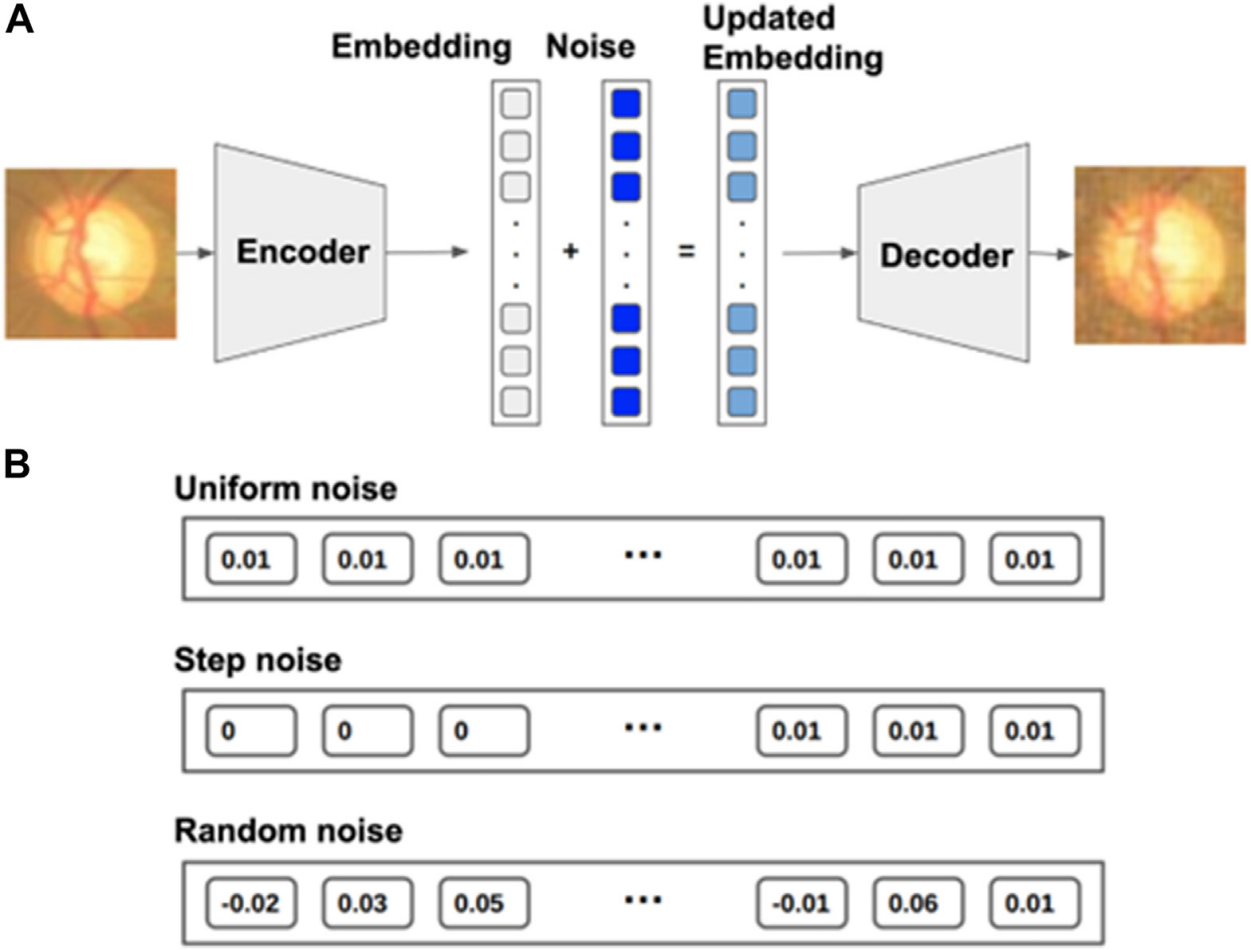
**A,** The process of producing synthetic images. **B,** representation of various types of noises used in this work.

**Figure 9:**
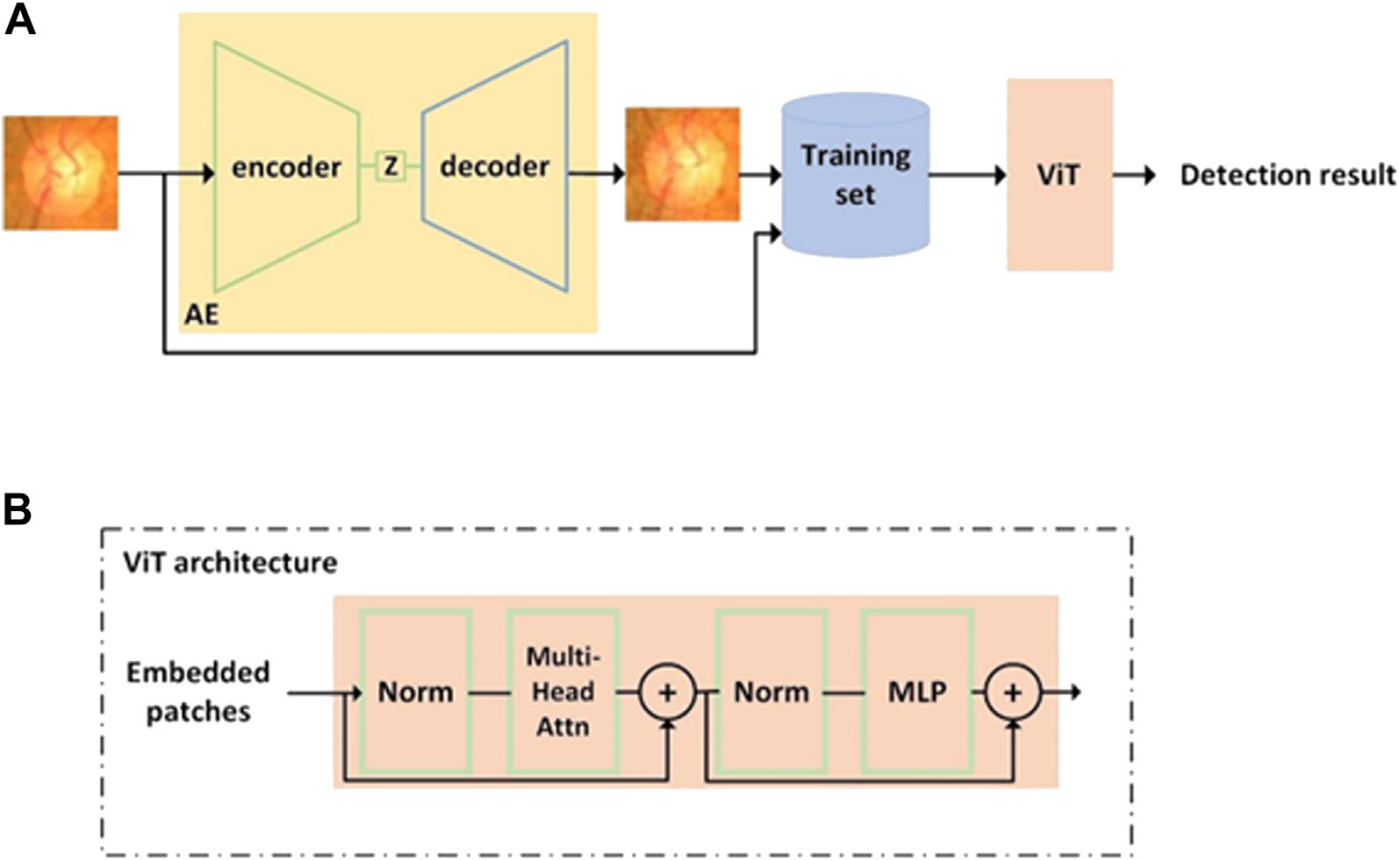
**A,** Overview of our approach for automated glaucoma detection. **B,** ViT architecture. ViT = vision transformer.

The key function of autoencoder is to produce high fidelity medical images for training set enhancement. We chose not to use generative adversarial networks^25^ because training such a network itself is data intensive, and techniques such as diffusion^27^ can lead to hallucinations and produce nonsensical images that are counterproductive for the training of healthcare AIs, as shown in Figure 7. Instead, we leveraged an autoencoder^28^ to generate synthetic images whereby controlled variations were introduced to real-world medical images (Fig 2). These variations go beyond simple rotations or the addition of noises. autoencoder first abstracts existing images to embeddings (encoding process), before adding transformations through a neural net. The altered embeddings are then used to generate high fidelity synthetic images of the original medical data (decoding process). The encoder and decoder are both multilayer ResNets^29^ and can be used to encode/decode any image. Their detailed neural net architectures are illustrated in Figure 3, Figure 4, Figure 5 and Fig 6. For the purposes of this study, we applied autoencoder to optic disc photos (Fig 8A) from publicly available databases. Each optic disc photo was used to generate 3 synthetic images by introducing different types of variations. A total of 100% of the resultant images were recognizable as optic disc photos (Fig 8B); one-third of these synthetic images were randomly selected to expand the training set by 100%, improving AI performance in the detection of glaucomatous optic neuropathy (Fig 9A).

### Autoencoder Enhancement of ViT-Based Glaucoma Detection

We tested the performance of autoencoder on 6 different publicly available training sets (Table 2) for glaucomatous optic neuropathy, ranging from a minimum of 101 images (Drishti-GS1) to a maximum of 800 images (REFUGE2), each prelabeled as positive/glaucomatous images versus negative/control images. The vision transformer was first trained on 80% of each dataset and evaluated on the remaining 20%, confirming performance levels similar to previous works with these datasets in the literature.^9^^,^^10^ Each database was then enhanced by synthetic images generated by autoencoder, leading to a 100% expansion of the training set (80% of each dataset). The same ViT model was then retrained using this autoencoder-enhanced dataset and evaluated on the same (all original, unenhanced) 20% validation subset of each database. Specific performance metrics are reported below, organized by database, in order from smallest dataset to largest.

**Table 2:** Characteristics of Publicly Available Datasets Used in this Study

Dataset	Description	Data Volume	
		Glaucoma	Non-Glaucoma
Drishti-GS1	Expert-selected images of glaucomatous and routine refractions obtained from patients aged 40–80 at Aravind Eye Hospital in India.	70	31
sjchoi86-HRF	Not available	101	300
RIM-ONE	Curated extraction from RIM-ONE V1, V2, and V3 of glaucomatous and healthy patients originating from 3 hospitals in Spain	173	312
ORIGA	Glaucomatous and randomly selected non-GC images from the cross-sectional population study in Singapore (SiMES). The study specifically focuses on Malay adults aged between 40 and 80	168	482
ACRIMA	Glaucomatous and normal images selected by experts in Spain based on clinical findings	396	309
REFUGE2	Random selection from glaucoma and myopia study cohorts in China (Zongshan Ophthalmic Center)	80	720

### Drishti-GS1

**Figure 10:**
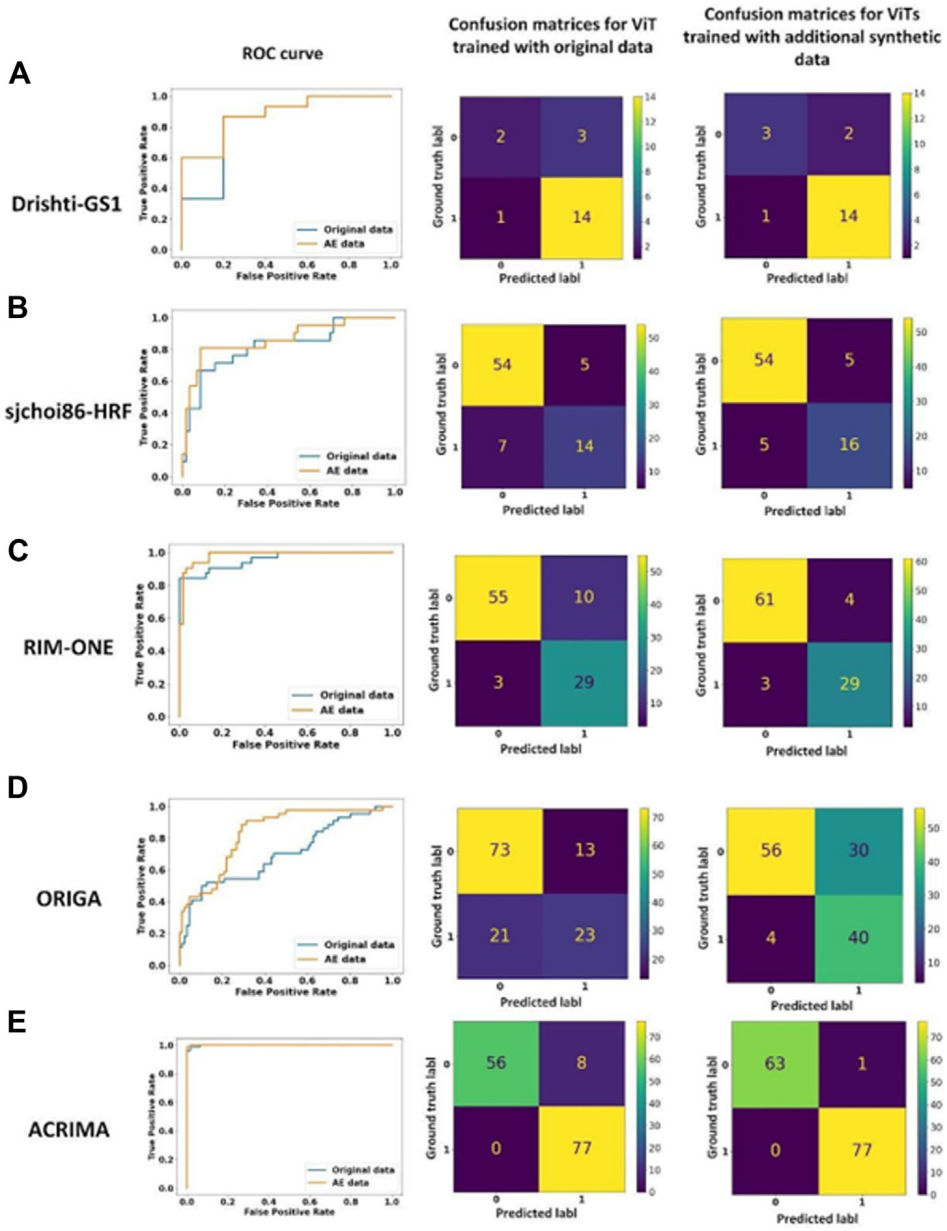
Results for 5 datasets: First column presents ROC curves for ViT trained with original data and ViT trained with additional synthetic data. Second column presents confusion matrix for ViT trained with original data. Third column presents confusion matrix for ViT trained with additional synthetic data. ROC = receiver operating characteristic; ViT = vision transformer.

The Drishti-GS1 dataset consists of 101 retinal fundus images, with 70 images representing positive/glaucoma samples and 31 images as negative/control samples. The vision transformer trained on Disghti-GS1 showed an area under the receiver operating characteristic curve (AUC) of 0.67, a sensitivity of 0.93, and a specificity of 0.40. When enhanced with 80 synthetic images (Drishti-GS1 + autoencoder), ViT performance improved to an AUC of 0.77, sensitivity of 0.93, and specificity of 0.60 (Fig 10 and Table 3).

**Table 3:** The Results for Drishti-GS1 Dataset

Dataset	Original Data Used Volume	Autoencoder Enhancement	AUC (95% CI)	Sensitivity	Specificity
Drishti-GS1	100%	False	0.67 (0.44, 0.97)	0.93 (0.79, 1.00)	0.40 (0.00, 1.00)
Drishti-GS1	100%	True	0.77 (0.47, 1.00)	0.93 (0.79, 1.00)	0.60 (0.00, 1.00)

AUC = area under the receiver operating characteristic curve; CI = confidence interval.

### sjchoi86-HRF

The sjchoi86-HRF dataset consists of 401 retinal fundus images, of which 101 are positive/glaucoma samples and 300 are negative/control samples. The vision transformer trained with sjchoi86-HRF yielded an AUC of 0.79, sensitivity of 0.67, and specificity of 0.92. When enhanced with 320 synthetic images (sjchoi86-HRF + autoencoder), ViT achieved an improved AUC of 0.84, sensitivity of 0.76, and specificity of 0.92 (Fig 10 and Table 4).

**Table 4:** The Results for sjchoi86-HRF Dataset

Dataset	Original Data Used Volume	Autoencoder Enhancement	AUC (95% CI)	Sensitivity	Specificity
sjchoi86-HRF	100%	False	0.79 (0.67–90)	0.67 (0.46–0.82)	0.92 (0.84–0.98)
sjchoi86-HRF	100%	True	0.84 (0.73–0.93)	0.76 (0.57–0.94)	0.92 (0.84–0.98)

AUC = area under the receiver operating characteristic curve; CI = confidence interval.

### RIM-ONE

RIM-ONE contains 485 retinal fundus images, of which 173 are positive/glaucoma samples and 312 are negative/control samples. The vision transformer trained with RIM-ONE showed an AUC, sensitivity, and specificity of 0.88, 0.91, and 0.85 respectively. When enhanced with 388 synthetic images (RIM-ONE + autoencoder), ViT was able to achieve an improved AUC, sensitivity, and specificity of 0.92, 0.91, and 0.94 respectively (Fig 10 and Table 5).

**Table 5:** The Results for RIM-ONE Dataset

Dataset	Original Data Used Volume	Autoencoder Enhancement	AUC (95% CI)	Sensitivity	Specificity
RIM-ONE	100%	False	0.88 (0.81–0.94)	0.91 (0.79–1)	0.85 (0.76–0.93)
RIM-ONE	100%	True	0.92 (0.86–0.97)	0.91 (0.79–1)	0.94 (0.88–0.99)

AUC = area under the receiver operating characteristic curve; CI = confidence interval.

### ORIGA

The ORIGA dataset comprises 650 retinal fundus images, with 168 images representing positive/glaucoma samples and 482 images serving as negative/control samples. The vision transformer trained on ORIGA performed with 0.69 AUC, 0.52 sensitivity, and 0.85 specificity. When enhanced with 520 synthetic images (ORIGA + autoencoder), ViT performance improved to an AUC of 0.78, sensitivity of 0.91, and specificity of 0.65 (Fig 10 and Table 6).

**Table 6:** The Results for ORIGA Dataset

Dataset	Original Data Used Volume	Autoencoder Enhancement	AUC (95% CI)	Sensitivity	Specificity
ORIGA	100%	False	0.69 (0.60–0.77)	0.52 (0.37–0.67)	0.85 (0.77–0.92)
ORIGA	100%	True	0.78 (0.71–0.84)	0.91 (0.82–0.98)	0.65 (0.54–0.75)

AUC = area under the receiver operating characteristic curve; CI = confidence interval.

### ACRIMA

The ACRIMA dataset comprises 705 retinal fundus images, with 396 images representing positive/glaucoma samples and 309 images representing negative/control samples. The vision transformer trained on ACRIMA showed an AUC of 0.94, a sensitivity of 1, and a specificity of 0.88. When enhanced with 564 synthetic training images (ACRIMA + autoencoder), ViT performance improved to an AUC of 0.99, sensitivity of 1, and specificity of 0.98 (Fig 10 and Table 7).

**Table 7:** The Results for ACRIMA Dataset

Dataset	Original Data Used Volume	Autoencoder Enhancement	AUC (95% CI)	Sensitivity	Specificity
ACRIMA	100%	False	0.94 (0.90–0.97)	1 (1.00–1.00)	0.88 (0.79–0.95)
ACRIMA	100%	True	0.99 (0.97–1)	1 (1.00–1.00)	0.98 (0.95–1.00)

AUC = area under the receiver operating characteristic curve; CI = confidence interval.

### Reduction in Training Data Requirement

**Figure 11:**
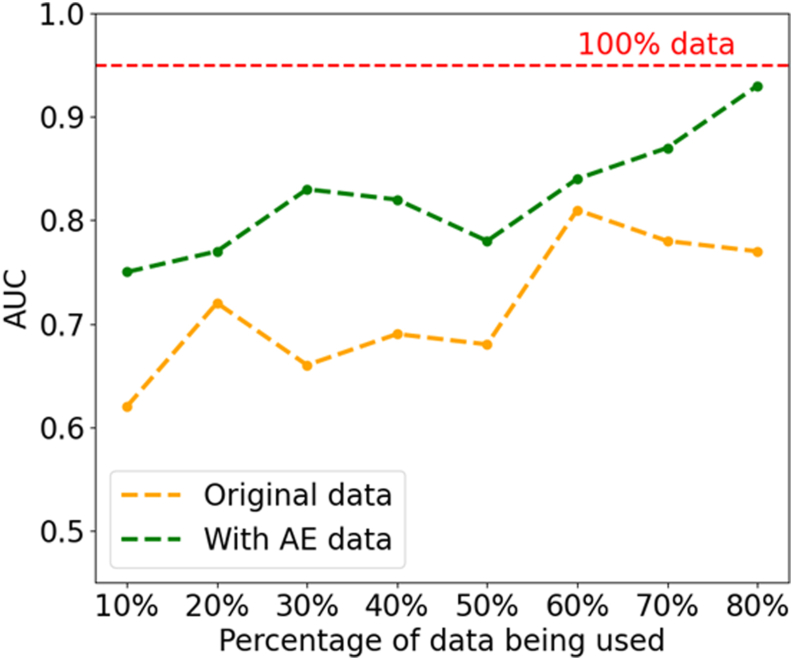
Results for REFUGE2 dataset: the AUC vs. percentage of original data being used in the training process. AUC = area under the receiver operating characteristic curve.

The largest publicly available database we explored is REFUGE2, which comprises 800 retinal fundus images, including 80 positive/glaucoma samples and 720 negative/control samples. The vision transformer trained using REFUGE2 resulted in an AUC of 0.95, sensitivity of 0.91, and specificity of 1.00. Given this excellent performance, we used this database to further assess the ability of our approach to reduce training data requirements. In Figure 11, “original data” underwent various image augmentation techniques, such as cropping, flipping, and randomization of image properties. To address data imbalance, we employed downsampling, implemented weighted loss, and used the best results as benchmarking for autoencoder enhanced dataset, “with AE data.” Figure 11 shows ViT performance using a random 10%, 20%, 30%, 40%, 50%, 60%, 70%, and 80% of the training subset of REFUGE2, resulting in AUCs of 0.62, 0.72, 0.66, 0.50, 0.68, 0.81, 0.78, and 0.77, respectively. When enhanced with synthetic images from autoencoder, ViT performance improved to AUCs of 0.75, 0.77, 0.83, 0.82, 0.78, 0.84, 0.87, and 0.93, respectively. It is worth noting that when using 80% of training data with synthetic images, the AUC is comparable to that of the full training dataset, suggesting that the introduction of autoencoder and the use of resultant synthetic images are able to reduce the amount of data required for a similar level of ViT performance by 20%.

## Discussion

Developing accurate deep learning models for image analyses requires vast quantities of high-quality training data. This poses a significant bottleneck for many fields of AI development, particularly in data-limited fields such as health care.^30^ Traditional techniques for data cultivation, such as data gathering (e.g., vehicles equipped with various sensors drive around to capture training data for autopilot), data mining (e.g., extraction of product purchasing preferences from existing sources such as social media), and collaborative data collection (e.g., pooling of pockets of data) are difficult to implement in medicine. Patient recruitment is challenging for all fields of clinical studies. There are also growing concerns that underprivileged social groups, who have more health issues but less access to health care, are underrepresented in training sets, leading to biases in the resultant AIs.^31^, ^32^, ^33^ Dedicated equipment is needed for medical testing and highly specialized expertise is required for annotation of all collected medical data. Consequently, only select institutions are endowed for medical data gathering and mining, and these efforts often result in sub-optimal datasets, frequently too small and/or too uniform for deep learning model training.^34^ Collaborations to pool pockets of data are actively being explored but face challenges, such as the lack of standard data formats and the need to protect sensitive and identifying patient information.^35^, ^36^, ^37^ Furthermore, the narrow nature of deep learning models necessitates datasets that are tailored to the particular research question; thus, traditional data cultivation approaches are often cost prohibitive and/or insufficient for health care AI model development.

In this report, we introduce a novel approach for training data expansion and enhancement that can be applied toward any vision-based AI model development. By producing synthetic training images using an autoencoder, we were able to improve ViT performance in glaucoma detection from optic disc photos across 6 public databases. Compared against state-of-the-art AUCs reported in the literature for the 5 smaller databases (0.77 for ACRIMA,^9^ 0.80 for Drishti-GS1,^9^ 0.73 for ORIGA,^10^ 0.91 for RIM-ONE,^10^ and 0.77 for sjchoiHRF^9^), autoencoder enhanced training sets led to notable ViT performance improvements in 3 of the 5 databases, and comparable performance in the remaining 2 databases (0.99 for ACRIMA, 0.77 for Drishti-GS1, 0.78 for ORIGA, 0.92 for RIM-ONE, 0.84 for sjchoiHRF). These results demonstrate that the proposed approach is more effective compared to state-of-the-art approaches in glaucoma detection. The greatest impact of autoencoder-generated synthetic data was observed in datasets ACRIMA, ORIGA, and sjchoi86-HRF, which range from 101 to 705 images. In these datasets, the enhanced ViT exhibited an increase in AUC of ≥ 0.05 compared to the ViT trained solely on the original dataset. The superiority of the enhanced ViT is further evident from the confusion matrix, where higher accuracy is observed compared to the ViT trained without the synthetic images. These results indicate that autoencoder generated synthetic data can effectively mitigate the issue of data scarcity and improve AI model accuracy beyond existing benchmarks toward advancing the state-of-the-art in automated glaucoma detection.

**Figure 12:**
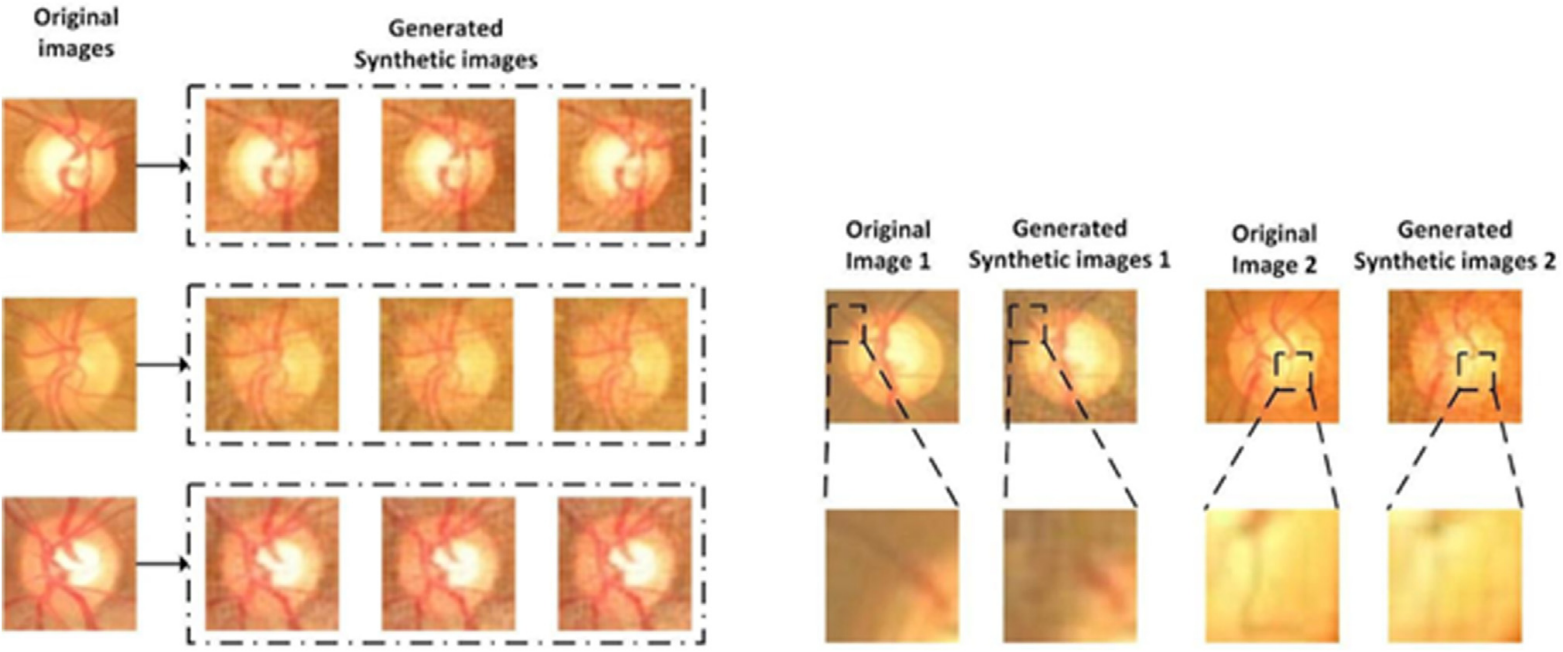
**A,** Visualization of autoencoder-generated synthetic images (**B**) variations introduced by autoencoder.

The improvements in ViT performance likely stem from enhanced diversity as well as quantity of the autoencoder-generated synthetic training sets. Figure 12 showcases representative synthetic images and highlights the variations introduced by the autoencoder. By contrasting the synthetic images with their original counterparts, we are able to see autoencoder added variations augmenting the diversity and richness of the training data. The inclusion of these synthetic images in the training set allows the ViTs to learn from a more extensive range of data, leading to a better understanding of the underlying patterns and features relevant to glaucoma detection, ultimately enhancing model performance.

One key feature of our reported AI system is generalizability to other types of image-based tasks. While we report here a medical usage case, the autoencoder was actually trained by nonmedical images. This allowed us to leverage existing image banks such as imageNet, which features a large volume of diverse images. Our results show that autoencoder trained with imageNet^37^ can successfully encode/decode medical images without any fine tuning, proving that the autoencoder is general, instead of overfitting to a small group of images. Together, these findings suggest that the methods proposed here can be used to effectively enhance AI model performance on any image-based small datasets.

It is interesting to note that while there is a loose trend of increased AUC with increasing training set size, this correlation does not hold true 100% of the time. Of the 6 public databases we evaluated, ORIGA (650 images) is bigger than RIM-ONE (485 images) and sjchoi86-HRF (401 images) but results in a worse AUC both in state-of-the-art results reported in the literature and in our own ViT. Possible reasons contributing to this discrepancy include differences in ground truth labeling, variations in data standards or image quality as well as other ophthalmic comorbidities that may be unknowingly featured in the training images. Although outside the scope of this report, investigations into why some datasets perform better than others may provide useful insights into how to build effective training databases.

We acknowledge a number of limitations in our study. Firstly, as with most nascent technologies, AI tools for health care are currently being developed largely in silico, shielded from the vast socioeconomic systems that greatly impact our health care system. As these technologies mature, they will need to be battle-tested in complex real-world ecosystems before deployment and require continuous vigilance leading up and beyond deployment. Technologically, our approach has only been tested on a single usage case to date. Whether the proposed framework can effectively enhance the performance of small datasets and reduce training data requirements in other fields remains to be characterized. In theory, the generalizability of our autoencoder, initially trained on a broad image corpus rather than a specialized medical imaging repository, suggests its applicability should extend beyond our current medical use case to other similarly unrelated tasks. Additionally, we report validation using 6 separate publicly available datasets, curated by different entities on distinct patient populations for varying purposes, thus showing the versatility of our method across multiple platforms. Secondly, the autoencoder is limited in its ability to produce highly diverse training sets. We intentionally favored this technology over other generative AI techniques for the autoencoder’s ability to generate synthetic images with superior fidelity by preserving fine details and maintaining image quality, but this strength conversely inhibits the autoencoder’s capacity for novel or unique output generation. We manually added elements of stochasticity through our methodology: for each real training image, we strategically generated 3 synthetic images but deployed only one of them, selected at random, to avoid overrepresentation of synthetic data and undue distortion of the training data landscape. Future works could explore various ratios of real versus generated synthetic versus included synthetic images to identify optimal conditions. Additionally, the diverse distribution of images generated by the diffusion technique has recently been shown to be tractable on medical image datasets with carefully designed conditions.^38^ Thus future frameworks could explore conditional diffusion models to further alleviate the data scarcity problem, generate more diverse and controllable synthetic images, and extend the application of the proposed framework to a broader range of medical applications.
